# Flux of signalling endosomes undergoing axonal retrograde transport is encoded by presynaptic activity and TrkB

**DOI:** 10.1038/ncomms12976

**Published:** 2016-09-30

**Authors:** Tong Wang, Sally Martin, Tam H. Nguyen, Callista B. Harper, Rachel S. Gormal, Ramon Martínez-Mármol, Shanker Karunanithi, Elizabeth J. Coulson, Nick R. Glass, Justin J. Cooper-White, Bruno van Swinderen, Frédéric A. Meunier

**Affiliations:** 1 Clem Jones Centre for Ageing Dementia Research, Queensland Brain Institute, The University of Queensland, Brisbane, Queensland 4072, Australia; 2 Menzies Health Institute Queensland, Griffith University, Gold Coast, Queensland 4222, Australia; 3 School of Biomedical Sciences, The University of Queensland, Brisbane, Queensland 4072, Australia; 4 Australian Institute for Bioengineering and Nanotechnology, The University of Queensland, Brisbane, Queensland 4072, Australia; 5 School of Chemical Engineering, The University of Queensland, Brisbane, Queensland 4072, Australia; 6 Materials Science and Engineering Division, CSIRO, Clayton, Victoria 3168, Australia; 7 Queensland Brain Institute, The University of Queensland, Brisbane, Queensland 4072, Australia

## Abstract

Axonal retrograde transport of signalling endosomes from the nerve terminal to the soma underpins survival. As each signalling endosome carries a quantal amount of activated receptors, we hypothesized that it is the frequency of endosomes reaching the soma that determines the scale of the trophic signal. Here we show that upregulating synaptic activity markedly increased the flux of plasma membrane-derived retrograde endosomes (labelled using cholera toxin subunit-B: CTB) in hippocampal neurons cultured in microfluidic devices, and live *Drosophila* larval motor neurons. Electron and super-resolution microscopy analyses revealed that the fast-moving sub-diffraction-limited CTB carriers contained the TrkB neurotrophin receptor, transiently activated by synaptic activity in a BDNF-independent manner. Pharmacological and genetic inhibition of TrkB activation selectively prevented the coupling between synaptic activity and the retrograde flux of signalling endosomes. TrkB activity therefore controls the encoding of synaptic activity experienced by nerve terminals, digitalized as the flux of retrogradely transported signalling endosomes.

## Abstract (web summary)

Signalling endosomes are known to be essential for neuronal survival. Here the authors show that, in cultured hippocampal neurons and live *Drosophila* larval motor neurons, neuronal activity increases the retrograde flux of signalling endosomes, and this coupling depends on TrkB activation.

## Graphical abstract

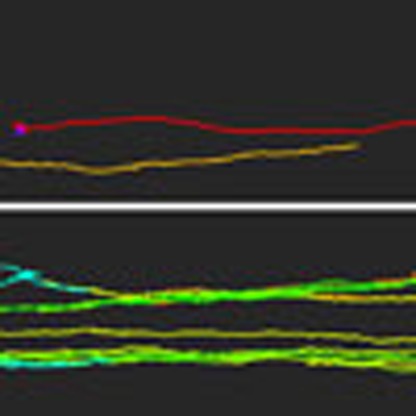

The development and survival of neurons are critically dependent on the transport of extracellular neurotrophic cues from the terminals to the cell bodies^1^. Signalling endosomes that specialize in delivering target-derived signalling survival cues via long-range retrograde trafficking^2^,^3^,^4^ play a critical role in neuronal survival, axon guidance and growth^5^. The retrograde trafficking of nerve growth factor and its receptor TrkA in signalling endosomes causes sustained activation of downstream pathways such as the mitogen-activated protein kinase (MAPK) and phosphoinositide 3-kinase pathways to promote the survival of innervating neurons^6^,^7^,^8^. Similarly, brain-derived neurotrophic factor (BDNF) and activated TrkB exert anti-apoptotic effects following retrograde transport to the soma^9^. Neuronal activation is known to promote TrkB receptor recruitment to the plasma membrane following ligand binding and internalization, thereby facilitating neurotrophic signalling triggered by autocrine- or paracrine-derived BDNF^10^,^11^,^12^.

Recent studies have revealed that signalling endosomes only contain a limited number of activated receptors, indicating that the survival signal may be derived from quantal units^6^,^13^. Hence the number of signalling endosomes, rather than the number of activated receptors per signalling endosome, determines the scale of the trophic signal reaching the soma. We therefore hypothesized that upregulating synaptic activity would lead to an increased number of signalling endosomes undergoing retrograde transport to ensure delivery of survival signals in neurons. Presynaptically endocytosed cholera toxin subunit-B (CTB), a highly efficient retrograde tracer in neurons, converges with the neurotrophin receptor p75^NTR^ at the level of early endosomes and throughout their axonal retrograde transport to the soma^1^,^14^,^15^,^16^. This suggested that CTB could be a useful probe for detecting retrograde signalling endosomes. Here we directly examine the relationship between retrograde signalling endosomes and neuronal activity, and analyse the morphology, number and dynamic characteristics of signalling endosomes generated at the nerve terminal using CTB.

## Results

### Activity promotes CTB uptake into endosomes and synaptic vesicles

**Figure 1:**
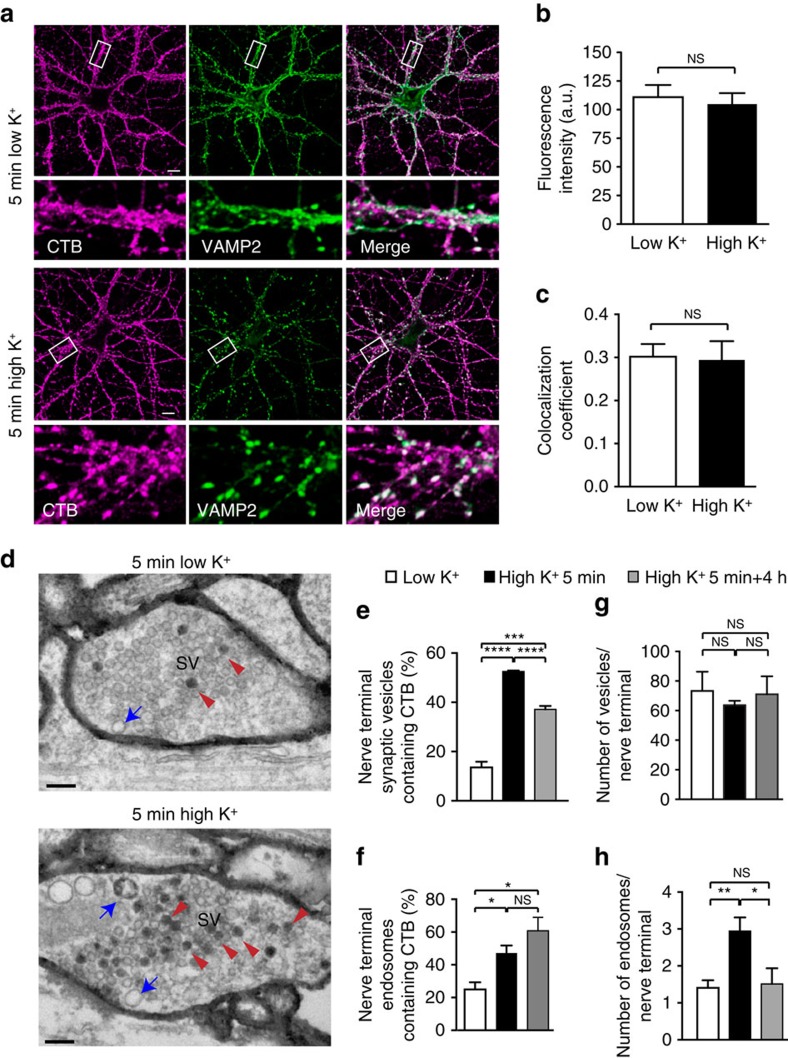
Analysis of CTB binding and endocytosis in primary hippocampal neurons. (**a**) Cultured hippocampal neurons were washed once with low K^+^ buffer then incubated with CTB-Af555 for 5 min in either low or high K^+^ buffer before fixation. Neurons were processed for immunocytochemistry using an anti-VAMP2 antibody. Scale abr, 10 μm. (**b**,**c**) Fluorescence intensity of CTB in regions of interest (ROIs) alone (**b**) or in co-localization with VAMP2 (Pearson’s coefficient) (**c**) were determined for each condition, and no significant difference in either analysis was observed between low and high K^+^ treatments. (mean±s.e.m., *n*=3 independent experiments, 10 cells (2 ROIs each) per experiment, Student’s *t*-test). (**d**) Cultured hippocampal neurons incubated with CTB-HRP (10 μg ml^−1^) for 5 min in either low or high K^+^ buffer before fixation. Cells were fixed, processed for DAB cytochemistry and imaged by electron microscopy. Compartments within the presynaptic terminal containing endocytosed CTB were identified using the DAB reaction product. Compartments were identified as vesicles (red arrowheads) or endosomes (blue arrows). Scale bar, 200 nm. (**e**–**h**) Cultured hippocampal neurons were treated as in **a**, then either fixed (5 min) or returned to growth medium for 4 h before fixation (5 min+4 h). Following processing, the number of small vesicles (<80 nm diameter) and endosomes (>80 nm diameter) per nerve terminal were quantified and the proportion of these compartments containing CTB was determined (mean±s.e.m., *n*=3–4 individual neuron preparations, >20 nerve terminals per preparation. *P<0.05, **P<0.01, ***P<0.001, ****P<0.0001, NS, not significant, Student’s *t*-test).

We hypothesized that the flux of signalling endosomes reaching the cell body following retrograde transport encodes a temporal signature of the level of activity occurring at the nerve terminals. To test this, we first examined the effect of increasing presynaptic activity on the uptake of the endocytic marker CTB into nerve terminals. Hippocampal neurons cultured for 14–16 days *in vitro* (DIV) were incubated with fluorescently tagged CTB (CTB-Af555) in low K^+^ (5.6 mM K^+^) or during depolarization by isotonic high K^+^ buffer (56 mM K^+^) for 5 min, then fixed and labelled for the synaptic vesicle protein, VAMP2/synaptobrevin2 (Fig. 1a). We found that CTB labelling was concentrated in puncta and showed significant overlap with VAMP2, consistent with our previous finding that CTB enters presynaptic nerve terminals^17^. However, quantification of the intensity of CTB labelling in resting and activated neurons revealed no significant difference (Fig. 1b). Furthermore, the co-localization of CTB and VAMP2 was unchanged following stimulation (Fig. 1c). These data confirm that CTB is recruited to the synaptic areas of axons, but fail to distinguish a difference between stimulated and unstimulated states.

One of the defining morphological features of the presynaptic nerve terminal is the high concentration of organelles within a very restricted spatial area, including synaptic vesicles and small endosomes in close proximity to the cell surface. Considering that the average diameter of a synaptic vesicle is ∼45 nm and that of a bulk endosome is ∼100 nm, and that a sizeable amount of CTB remains bound to the cell surface (Fig. 1d), we considered the possibility that identifying differences in endocytosis and localization of CTB could be beyond the resolution limit of the confocal microscope. We therefore further examined the distribution of CTB within the synapse by electron microscopy, under resting (low K^+^) and activated (high K^+^) conditions. In contrast to our confocal microscopy results, we found that the proportion of small vesicles (40–80 nm) within the presynaptic terminal that contained endocytosed CTB was significantly higher in the stimulated condition than in the resting state (Fig. 1e). Similarly, there was a significant increase in the number of larger electron-lucent compartments (>80 nm diameter) containing CTB (Fig. 1f), which are likely to be activity-dependent bulk endosomes triggered by depolarization^18^. No difference was detected in the amount of CTB present at the cell surface. Likewise, the total number of small vesicles did not change with stimulation (Fig. 1g). However, we observed an activity-dependent increase in the number of bulk endosomes (Fig. 1h). To investigate the fate of the CTB-positive vesicles and bulk endosomes post-activity, we analysed the number of labelled compartments in the nerve terminals following pulse labelling with CTB for 5 min in high K^+^, followed by a 4 h chase (replacement of buffer with normal growth medium). Although the number of endosomes returned to control levels following the chase, suggesting that the rate of endocytosis had decreased, the proportion of labelled bulk endosomes remained high at the end of the chase period, consistent with an ongoing increased basal rate of clathrin-mediated endocytosis of CTB in stimulated neurons (Fig. 1f). In contrast, whereas the total number of small synaptic vesicles in the nerve terminals remained unchanged (Fig. 1g), the proportion of synaptic vesicles that contained CTB was significantly decreased after 4 h (Fig. 1e), suggesting that these compartments were being specifically targeted for removal from the nerve terminal during the chase period, potentially through retrograde transport to the soma.

### Synaptic activity increases the flux of CTB retrograde carriers

**Figure 2:**
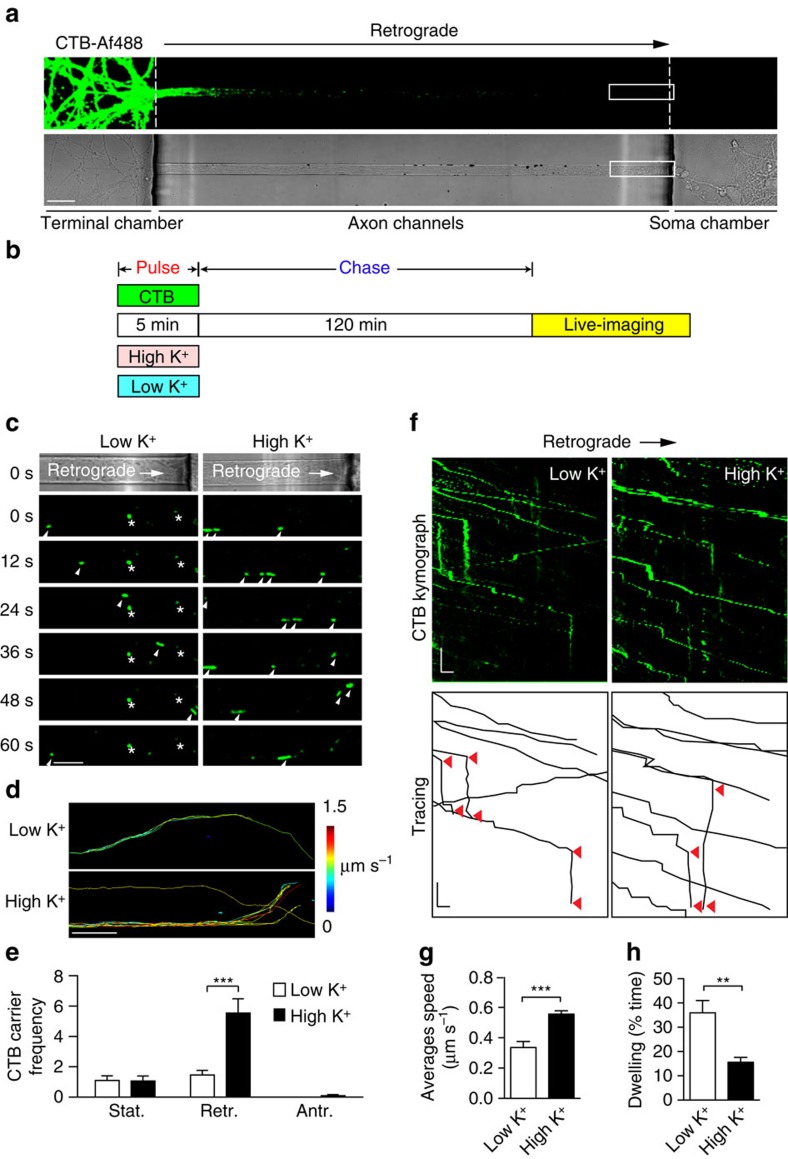
The flux of retrograde CTB carriers is activity-dependent in cultured rat hippocampal neurons. (**a**) CTB-Af488 labelling was performed in nerve terminals of hippocampal neurons cultured in microfluidic devices. CTB retrograde trafficking was captured in the observation window along the axon channels (boxed). Scale bar, 20 μm. (**b**) Hippocampal neurons cultured in microfluidic chambers were pulsed at 37 °C for 5 min with CTB in low- or high-K^+^ buffers, followed by wash-off of the pulse solutions before a chase in the original culture medium for 2 h. They were then live-imaged with confocal microscopy. (**c**) Time-lapse images of CTB carriers of axons as described in **a**; arrowheads delineate the retrograde carriers. Asterisks indicate stationary carriers. Scale bar, 10 μm. (**d**) Imaris tracing of CTB tracks in representative movies (colour-coded for average speeds of 0–1.5 μm s^−1^). Scale bar, 10 μm. (**e**) Number of retrograde CTB carriers after high or low K^+^ treatment. (**f**) Representative kymographs of CTB carriers along a single axon. Tracing demonstrates track displacement and static periods (dwelling, red arrowheads). *x*— Scale bar, 5 μm; *y*— Scale bar, 20 s. (**g**,**h**) The average track speed (**g**) and dwelling time (**h**) were determined in low and high K^+^. (mean±s.e.m., *n*=15 and 20 channels for low and high K^+^ respectively, data from three independent experiments, Student’s *t*-test, **P<0.01, ***P<0.001).

Our observation that the number of small vesicles containing CTB decreased over time suggested that a subset of the generated endocytic organelles were destined for retrograde transport, and thus could be precursors to signalling endosomes delivering trophic signals to the soma. In order to test this hypothesis, we directly examined the effect of a short pulse of stimulation on the rate of retrograde CTB carriers along the unidirectional axon bundles of hippocampal neurons cultured in microfluidic devices (Fig. 2a). Fluorescently tagged CTB was added to the nerve terminal chamber for 5 min in either low K^+^ or high K^+^ buffer (pulse). During this step, a hydrostatic pressure difference was introduced between the nerve terminal chamber and the soma chamber to allow microfluidic flow, thereby restricting the CTB-containing medium from diffusing into the soma chamber^16^,^19^. After pulse-labelling, the cells were washed, returned to growth medium and incubated for a further 2–4 h (chase; Fig. 2b). To visualize the retrograde transport of CTB, regions of the microfluidic channel proximal to the cell soma chamber were imaged by time-lapse confocal microscopy. The movies of fluorescently labelled CTB carriers undergoing retrograde axonal transport were analysed for kinetic parameters such as frequency, speed and dwell time. In the axons of resting neurons (low K^+^), we observed a low number of CTB carriers undergoing retrograde movement, with most appearing stationary during the course of imaging (Fig. 2c,d; Supplementary Movie 1). No significant anterograde mobility was detected, although transient steps were occasionally observed (Fig. 2e). Stimulation resulted in a significant increase in the flux of CTB retrograde carriers (Fig. 2c–e; Supplementary Movie 2). Detailed analysis revealed not only a threefold increase in the number of CTB-positive retrograde carriers but also an increase in their average speed (Fig. 2e–g), concomitant with a decrease in the proportion of time that individual carriers spent dwelling (Fig. 2h). This is illustrated in the kymograph of CTB carriers (Fig. 2f; quantified in Fig. 2h). Importantly, the number of slow-moving CTB carriers significantly decreased following stimulation (Supplementary Fig. 1a, ‘stationary’). Conversely, we found a significant increase in the number of fast-moving carriers with an instantaneous speed of 1.0–2.5 μm s^−1^ (Supplementary Fig. 1a, ‘fast’)^20^. This suggests that the number of fast carriers (instant speed >0.5 μm s^−1^) increases at the expense of stationary carriers (instant speed <0.5 μm s^−1^) in response to stimulation (Supplementary Fig. 1b).

Presynaptic endocytosis can be up- or downregulated in response to different types of stimuli. For instance, BDNF exerts an inhibitory effect^21^, whereas KCl depolarization or more physiological generation of action potentials increases endocytosis^22^. To explore whether different stimuli affect the flux of retrograde carriers, we used either exogenously applied BDNF or bicuculline, a GABA_A_ (γ-aminobutyric acid) receptor blocker, which induces bursts of action potentials in cultured neurons^23^. We first measured the number of retrograde CTB carriers elicited in response to bicuculline (20 μg ml^−l^) treatment. We found that, similar to high K^+^ depolarization, 1 h treatment with bicuculline led to a significant increase in the retrograde flux of CTB carriers (Supplementary Fig. 1c–e). In contrast, applying BDNF (50 ng ml^−l^) did not increase the flux of retrograde carriers (Supplementary Fig. 1c–e). These results demonstrate that raising synaptic activity alone controls the flux of retrograde CTB carriers and that exogenously applied BDNF has no such effect.

**Figure 3:**
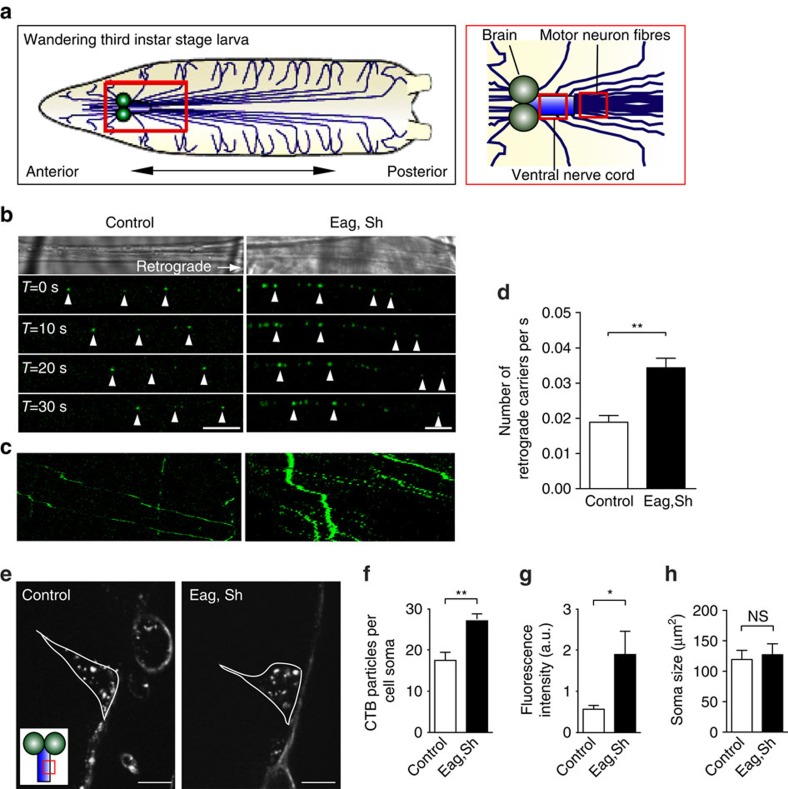
CTB axonal retrograde carrier flux is controlled by synaptic activity in *D. melanogaster* motor neurons. (**a**) Schematic figure of a *Drosophila* third instar stage larva; the central nervous system is detailed on the right. (**b**) Control and *eag, Sh* larva preparations were incubated with CTB-Af488 for 2 h, followed by time-lapse imaging by confocal microscopy. Arrowheads indicate the retrograde movement of the CTB-positive carriers along the motor neuron fibres in successive frames. Scale bar, 5 μm. (**c**) Kymographs generated from the same time-lapse movies shown in **b**. (**d**) Quantitative analysis of the flux of CTB-positive retrograde carriers in control, and *eag, Sh Drosophila* larva motor axons. (mean±s.e.m., *n*=15 and *n*=14 for control and *eag, Sh*, respectively, data from four independent preparations for each condition, **P<0.01, Student’s *t*-test). (**e**) Accumulation of retrogradely transported CTB-positive cargoes in motor neuronal cell bodies located in the ventral nerve cord of control and *eag, Sh* larvae after 4 h incubation with CTB-Af555. Inset indicates the location of the imaged cell bodies. Scale bar, 10 μm. Quantification of accumulated CTB-positive structures as number of structures per cell body (**f**) and as fluorescence intensity (**g**). The size of the motor neuronal soma was quantified in (**h**). (mean±s.e.m., *n*=9 and *n*=10 for control and *eag, Sh*, respectively, data from four independent preparations, *P<0.05, **P<0.01, NS, not significant, Student’s *t*-test).

We next examined whether raising synaptic activity also promotes an increase in the flux of CTB retrograde transport *in vivo*, using live *Drosophila melanogaster* larval preparations^24^. In addition to allowing us to measure the effect of activity on retrograde carrier trafficking in a complementary model system, this approach also facilitated tracing the effect of presynaptic activity on the complete transport of CTB from the neuromuscular junction to the soma located in the central nervous system of the larvae (Fig. 3a). In the fibers of neurons from wild-type larvae we could detect a low, albeit consistent, rate of CTB-positive retrograde carriers (Fig. 3b,c). To analyse the flux of retrograde carriers under conditions of high activity, we employed transgenic larvae expressing dominant-negative forms of ether-à-go-go (Eag) and Shanker (Sh) voltage-gated potassium channels in motor neurons, which upregulate synaptic transmission. Consistent with our data in primary hippocampal neurons, we found that increased presynaptic activity at the neuromuscular junction of the *eag, Sh* mutant larvae also resulted in an increase in the frequency of retrograde CTB carriers (Fig. 3b–d). Furthermore, the net transport of CTB could be quantified by measuring the accumulation of fluorescent CTB in the somata located in the ventral nerve cord (Fig. 3e–h). These data confirmed that presynaptic activity controls the transport of CTB retrograde carriers as well as their accumulation in the cell bodies located in the central nervous system.

### Most retrograde CTB carriers are sub-diffraction limited

**Figure 4:**
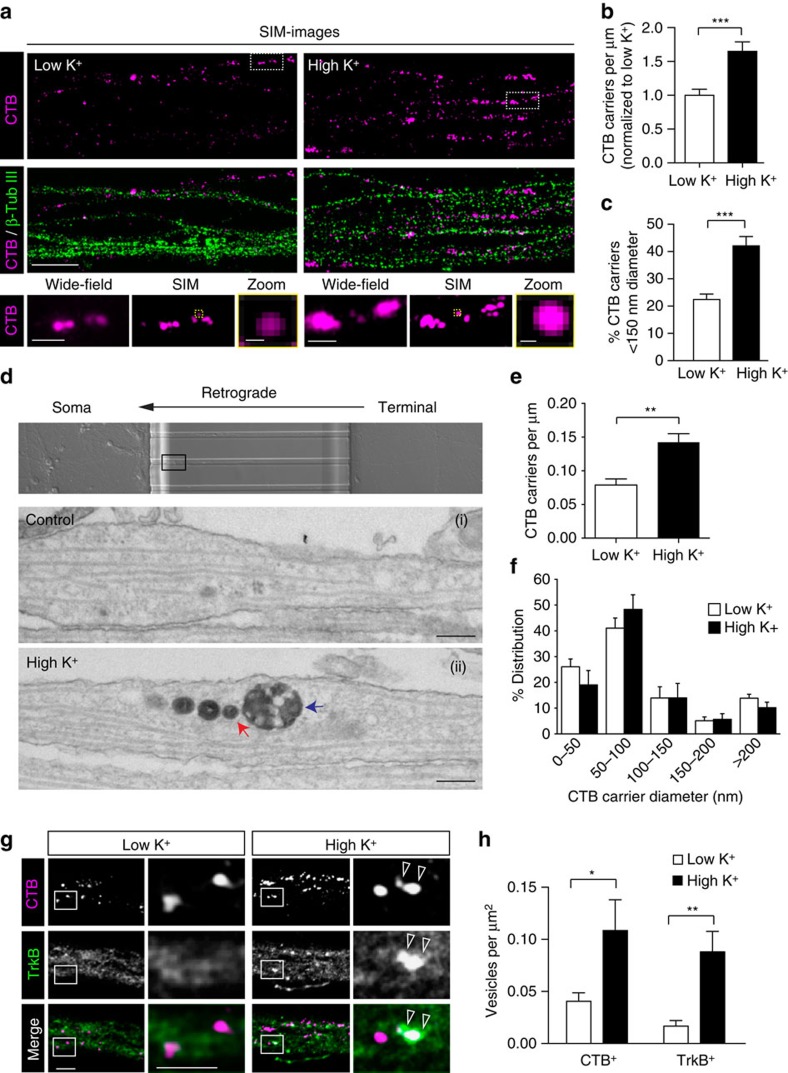
CTB retrograde carrier flux is composed of small aligned vesicular compartments. (**a**) Dual-colour SIM on axon channels of microfluidic chambers labelled with CTB under either low K^+^ (left) or high K^+^ (right) conditions, β-tubulin III was used to label the axon fibers. Scale bar, 5 μm. Boxes are magnified in the lower panels; original wide-field images are compared with the SIM images of the same region (Scale bar, 1 μm), and individual CTB carriers are further magnified in yellow boxes (Scale bar, 100 nm). The resolution is ∼100 nm. Quantification of total (**b**) and small CTB carriers (diameter<150 nm) (**c**) from SIM images (Mander’s coefficient, mean±s.e.m., *n*=27 for low K^+^, *n*=31 for high K^+^, ***P<0.001, data from four independent preparations, Student’s *t*-test). (**d**) Electron microscopy of axons from neurons pulse-chased with CTB-HRP as described in **a**. Small vesicular carriers—red arrows, multivesicular body—blue arrow. Scale bar, 200 nm. (**e**) Quantification of the number of carriers per μm neurite length per axon channel. (mean±s.e.m., *n*=7 (low K^+^) or 16 (high K^+^) channels from two independent neuron preparations. **P<0.01) (**f**) Size distribution of CTB carriers in low K^+^ and high K^+^ stimulated cells (*n*=3 independent neuron preparations). (**g**) Confocal microscopy of axons from neurons pulse-chased with CTB as described in (**a**). Endogenous TrkB immunostaining is shown in green, CTB vesicles that co-localized with TrkB are indicated with arrowheads. Scale bar, 5 μm. (**h**) Quantification of CTB-positive (CTB^+^) and TrkB-positive (TrkB^+^) carriers after low K^+^ or high K^+^ treatments (*n*=10 and 11 for high K^+^ and low K^+^, *P<0.05, **P<0.01, data from two independent preparations; NS, not significant, Student’s *t*-test).

As many of the carriers identified in stimulated neurons by confocal microscopy appeared elongated and possibly tubular (Fig. 2c), as previously described for signalling endosomes that contain BDNF^6^, we next investigated the nature of the retrograde CTB carriers in hippocampal neurons using higher-resolution techniques, including electron microscopy and structured illumination microscopy (SIM). The 82 nm resolution of SIM allowed us to reveal that elongated retrograde compartments identified by wide-field resolution were often composed of multiple small, round vesicles containing CTB, with a diameter <150 nm (Fig. 4a). Consistent with our previous data, the number of both elongated CTB carriers under confocal resolution (Fig. 4g,h) and small CTB carriers (diameter <150 nm) visualized by SIM increased significantly following stimulation (Fig. 4b,c). These results suggested that the elongated signalling endosomes could actually be composed of several small and tightly apposed vesicles that move together along the retrograde route.

We therefore used electron microscopy to further resolve the morphology of these retrograde carriers (Fig. 4d), revealing a significant increase in the number of retrograde CTB carriers in axons following stimulation (Fig. 4e), consistent with our previous observations at the presynapse (Fig. 1d–h). Although a proportion of these carriers were endosomal compartments and multivesicular bodies (Fig. 4d (ii), blue arrow), a high proportion of the retrograde carriers were small (diameter<150 nm) vesicular compartments (Fig. 4d (ii), red arrow) in the axon of both resting and stimulated neurons, as quantified in Fig. 4f. Interestingly, in many cases these vesicles were aligned in rows (Fig. 4d (ii)), whereas no obvious tubular CTB carriers were detected. Altogether, these data suggest not only that small vesicles comprise the major component of the retrograde flux of signalling endosomes but also that the tubular-shaped signalling endosomes detected by confocal microscopy could in fact be composed of small vesicles that align and move together.

### Activity-dependent localization of TrkB to CTB carriers

**Figure 5:**
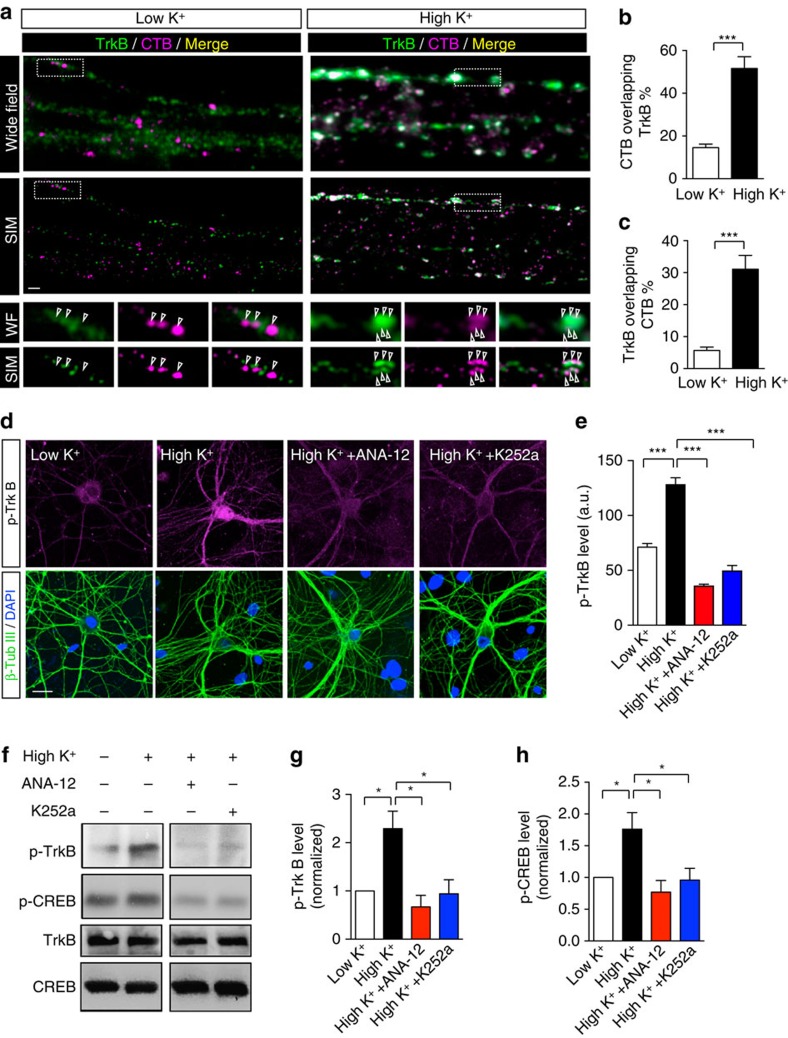
Activation of TrkB controls the activity-induced increase in CTB retrograde flux. (**a**) Representative wide-field and SIM images of axons in microfluidic chambers that were labelled with CTB (magenta) under either low K^+^ (left) or high K^+^ (right) conditions. TrkB antibody (green) was used to label endogenous TrkB receptors. Boxed regions magnified in the lower panels, and aligned CTB vesicles are pointed out with arrowheads. Scale bar, 1 μm. (**b**,**c**) Quantification of the ratio of CTB overlapping with TrkB (**b**), and the ratio of TrkB overlapping with CTB (**c**) from SIM images. (Mander’s coefficient, mean±s.e.m., *n*=39 (low K^+^) or *n*=46 (high K^+^) channels from three independent neuron preparations. ***P<0.001, Student’s *t*-test). (**d**) Activation of th TrkB pathway was examined with an antibody against phosphorylated Tyr^707/706^ of TrkB receptors (top panels). Pretreatment with 0.5 μM ANA-12 or 100 nM K252a for 30 min significantly inhibited the high K^+^-induced p-TrkB increase; β-tubulin III and DAPI (bottom panels) were used to show the density of neuron layers. Scale bar, 20 μm. (**e**) Quantification of **d**. (mean±s.e.m., *n*=29 (low K^+^) or 30 (high K^+^) channels from three independent neuron preparations. ***P<0.001, Student’s *t*-test). (**f**) Western blot of DIV 14 hippocampal neurons pretreated with 0.5 μM ANA-12 or 100 nM K252a for 30 min showing the same abolition of the high K^+^ induced increase in p-TrkB and phosphorylated CREB protein (p-CREB), which represented the downstream response; total TrkB and CREB protein levels were used as controls. (**g**–**h**) Quantification of **f**. (mean±s.e.m., *n*=3, data from three independent cultures, *P<0.05, Student’s *t*-test).

CTB is an excellent retrograde marker of endocytosis^25^,^26^ and co-localizes extensively with the signalling endosome components p75^NTR^ and BDNF^1^,^27^,^28^. To test whether it could be used as a marker for retrograde signalling endosomes, we analysed the co-localization of retrograde CTB with the BDNF receptor TrkB, a component of signalling endosomes^9^,^29^. We first confirmed that the endogenous TrkB level was not affected by CTB labelling (Supplementary Fig. 2a,b). Following a 5 min pulse of high K^+^ and a 2–4 h chase, we detected many large, tubular-shaped TrkB compartments, which extensively overlapped with CTB retrograde carriers (Fig. 4g,h). These were not detectable in unstimulated neurons. To confirm that the TrkB-positive compartments also contained CTB, we further resolved the localization of CTB and TrkB by SIM, which could distinguish the sub-diffractional compartments that appear ‘tubular’ by confocal microscopy. In agreement with our confocal data, we identified a significant increase in the number of TrkB carriers that co-localized with CTB carriers following high K^+^ and a 2 h chase, compared with the low-K^+^-treated neurons (Fig. 5a). The proportion of CTB-positive vesicles overlapping with TrkB-positive compartments increased from 14.5±1.7% in low K^+^ to 51.6±5.5% in high K^+^ (Fig. 5b). These data suggest that CTB and TrkB are either taken up or sorted into the same structures that undergo retrograde transport. Importantly, 31.1±4.3% of the TrkB-positive compartments co-localized with CTB (Fig. 5c), demonstrating that CTB indeed labels a large number of the signalling endosomes that are retrogradely transported^9^,^29^.

### TrkB activation couples activity with flux of CTB carriers

Neuronal activity upregulates the tyrosine kinase activity of TrkB, causing phosphorylation at tyrosines 707/706, located at the autophosphorylation site is required for further activation of TrkB^30^. Phosphorylated TrkB transduces intracellular signals to nuclear targets in the soma following retrograde trafficking in signalling endosomes^9^,^29^. To determine whether CTB-labelled retrograde endosomes correspond to signalling endosomes, we examined whether inhibiting TrkB activation prevented the increase in CTB retrograde flux. We used two different inhibitors of TrkB activity: ANA-12 a specific TrkB antagonist that prevents TrkB auto-phosphorylation by BDNF^31^, and K252a, which blocks tyrosine kinase activity, including that of TrkB^32^. Using an anti-phospho-Tyr706/707 (p-TrkB) antibody, we found that a pulse of 5 min high K^+^ depolarization induced a transient increase in the p-TrkB level, with a peak at 5 min (Supplementary Fig. 2c). In each subsequent western blot, the 5 min time point was therefore used. We also found an increase in the level of p-CREB, which occurs downstream of TrkB activation (Fig. 5d–h; Supplementary Figs 2d and 4). However, when neurons were pretreated with ANA-12 (0.5 μM) or K252a (100 nM) for 30 min before a 5 min depolarization pulse, the transient increase in p-TrkB was abolished (Fig. 5d,e). These data demonstrate that depolarizing stimulation can activate TrkB as previously reported^10^,^33^, and that both TrkB inhibitors can prevent such activation^31^,^32^.

**Figure 6:**
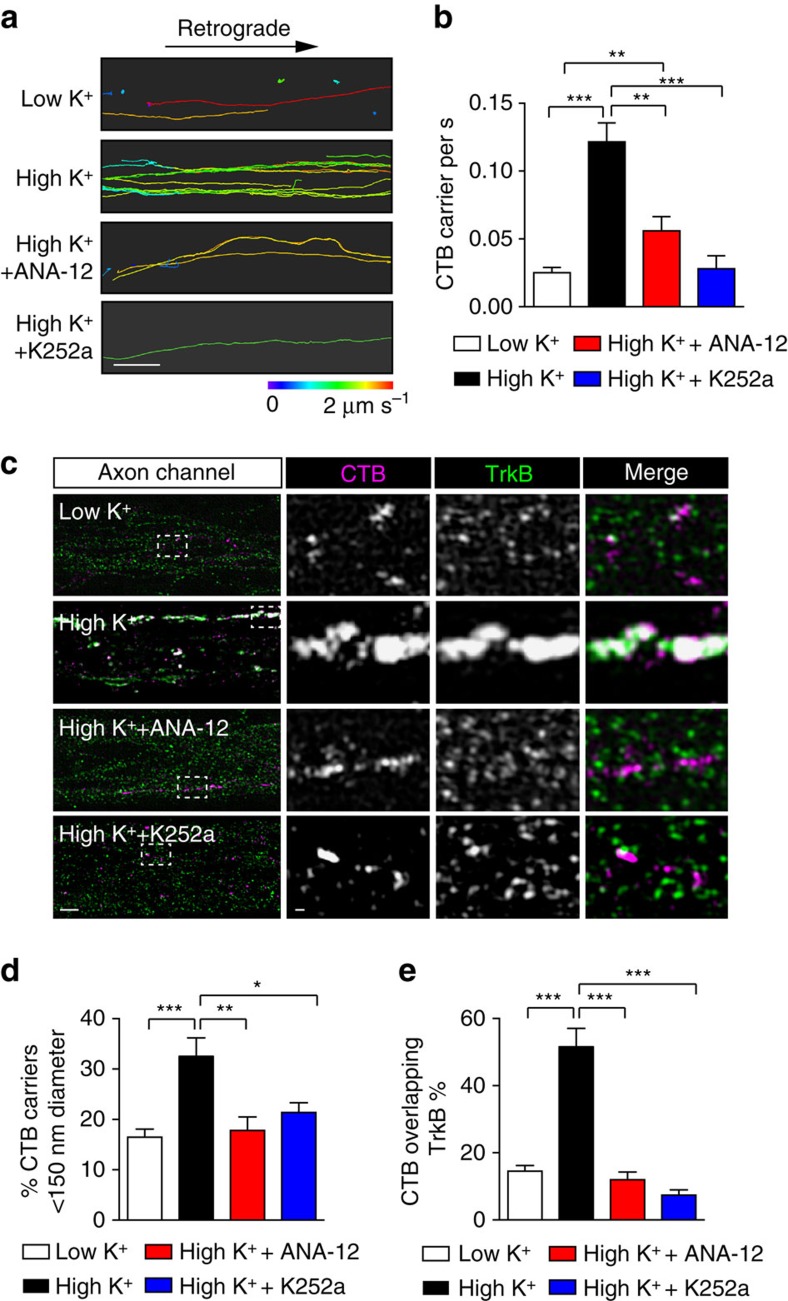
Pharmacological inhibition of TrkB activation abolishes the activity-dependent increase in CTB carrier flux undergoing retrograde axonal transport. (**a**) Representative images of CTB retrograde flux inside the axon channels of hippocampal neurons pretreated with 0.5 μM ANA-12 or 100 nM K252a for 30 min before high K^+^ pulse-chase. Neurons pulse-chased with low or high K^+^ alone were used as controls. Time-lapse images were tracked with Imaris software, and the tracks shown are colour-coded based on the average speed. Scale bar, 10 μm. Both inhibitors significantly abolished thehigh K^+^ stimulation-induced CTB flux, as reflected by the decreased number of CTB carriers trafficking through the axon channels per second as quantified in **b**, (mean±s.e.m., *n*=78 (low K^+^), 81 (high K^+^), 83 (high K^+^+ANA-12) and 45 (high K^+^+K252a) tracks, data from 3 independent neuron preparations, *P<0.05, **P<0.01,***P<0.001, Student’s *t*-test). (**c**) Representative SIM images of axons in microfluidic chambers. Neurons were labelled with CTB (red) under either low K^+^ or high K^+^ conditions. In the bottom groups, neurons were pretreated with ANA-12 or K252a for 30 min before high K^+^ stimulation. TrkB antibody (green) was used to label endogenous TrkB receptors, Scale bar, 2 μm. Boxed regions are magnified in the right panels, Scale bar, 200 nm. (**d**,**e**) Both inhibitors significantly reduced the number of small CTB carriers with a diameter <150 nm (**d**), as well as the ratio of CTB overlapping with TrkB as quantified in (**e**). (Mander’s coefficient, mean±s.e.m., *n*=39 (low K^+^), 46 (high K^+^), 32 (high K^+^+ANA-12) and 25 (high K^+^+K252a) tracks, data from three independent neuron preparations, *P<0.05, **P<0.01,***P<0.001, Student’s *t*-test).

As TrkB activation is required to generate signalling endosomes^10^,^33^, we next examined whether the activity-induced retrograde flux of CTB-labelled endosomes was affected by TrkB inhibition. Hippocampal neurons cultured in microfluidic chambers were incubated with dimethyl sulfoxide, ANA-12 or K252a for 30 min before undergoing a pulse-chase incubation, after which retrograde CTB carriers were imaged by confocal microscopy. Regions within an axon channel were imaged live for CTB carriers, and each trace was colour-coded for average speed (Fig. 6a). ANA-12 or K252a pretreatment effectively abolished the activity-dependent increase in CTB retrograde flux, as shown by a decrease in both the frequency (Fig. 6a,b; Supplementary Movies 3 and 4) and instant speed (Supplementary Fig. 3a) of the CTB carriers. In addition, the number of fast retrograde carriers was significantly decreased after TrkB inhibition, compared with the high K^+^-stimulated group (Supplementary Fig. 3b). Consistent with this, the number of stationary carriers did not decrease in response to stimulation as a result of TrkB inhibition (Supplementary Fig. 3b). These data reveal that preventing activity-induced TrkB activation abolishes the coupling between synaptic activity and CTB retrograde endosome flux.

To further test whether CTB was present in signalling endosomes that eventually delivered TrkB signals to the soma, we also performed SIM microscopy and assessed the proportion of CTB endosomes that carried TrkB receptors along the axons following inhibition of TrkB. We found that, compared with the group pretreated with dimethyl sulfoxide, the flux of CTB carriers elicited by a stimulatory pulse was significantly decreased in both ANA-12- and K252a-pretreated cells (Fig. 6c,d). Consistent with this, the proportion of CTB carriers overlapping with TrkB was also significantly decreased, and the level of co-localization between CTB and TrkB in the inhibitor-treated cells was reduced to that of the unstimulated cells treated with low K^+^ (Fig. 6c,e). These data demonstrate that transient activation of TrkB receptors is required to control the flux of retrograde signalling endosomes, which contain both CTB and TrkB receptors.

**Figure 7:**
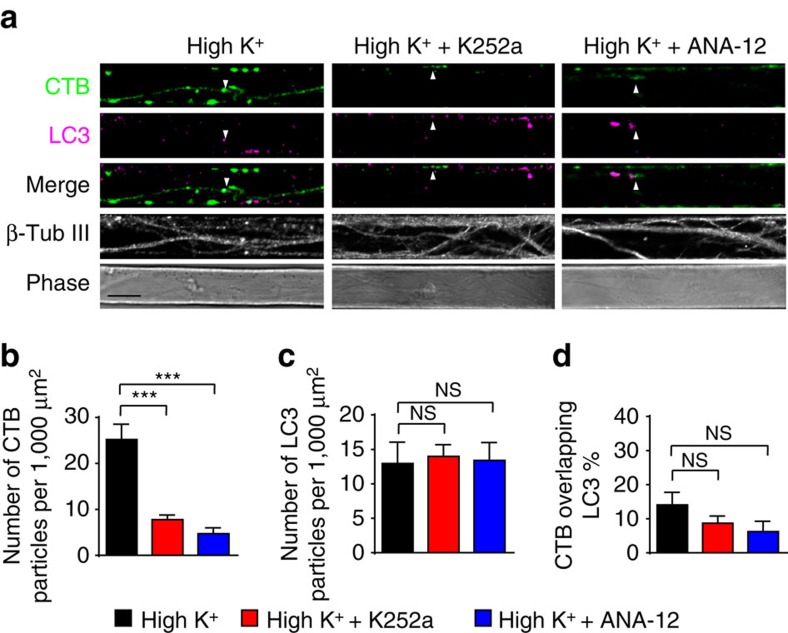
Pharmacological inhibition of TrkB activation has no effect on LC3-positive autophagosomes. (**a**) Representative images of axons in microfluidic chambers labelled with CTB under high K^+^ or pretreated with ANA-12, K252a for 30 min. TrkB antibody was used to label endogenous TrkB receptors, and co-localization is indicated with arrowheads. Scale bar, 10 μm. (**b**–**d**) Both inhibitors significantly reduced the number of CTB carriers (**b**) but had no significant effect on the level of the autophagosome marker LC3 in the same region of interest (**c**). The ratio of CTB overlapping with LC3 was also not affected (**d**). (Mander’s coefficient, mean±s.e.m., *n*=39 (high K^+^), 39 (high K^+^+ANA-12) and 38 (high K^+^+K252a) channels, data from two independent neuron preparations, ***P<0.001, NS, no significant difference; Student’s *t*-test).

As we have previously demonstrated that synaptic activity also increases the flux of retrograde autophagosomes in hippocampal neurons^16^, we tested the ability of TrkB inhibition to prevent the retrograde flux of autophagosomes labelled with LC3 (Fig. 7a). Increased numbers of both LC3 and CTB axonal carriers were observed following increased synaptic activity, as previously shown^16^. However, only the flux of CTB carriers was sensitive to pharmacological TrkB inhibition (Fig. 7b), with the number of retrograde autophagosomes remaining unaffected (Fig. 7c,d). This demonstrates that TrkB selectively controls the coupling between synaptic activity and the flux of signalling endosomes undergoing retrograde axonal transport^4^,^31^,^32^.

**Figure 8:**
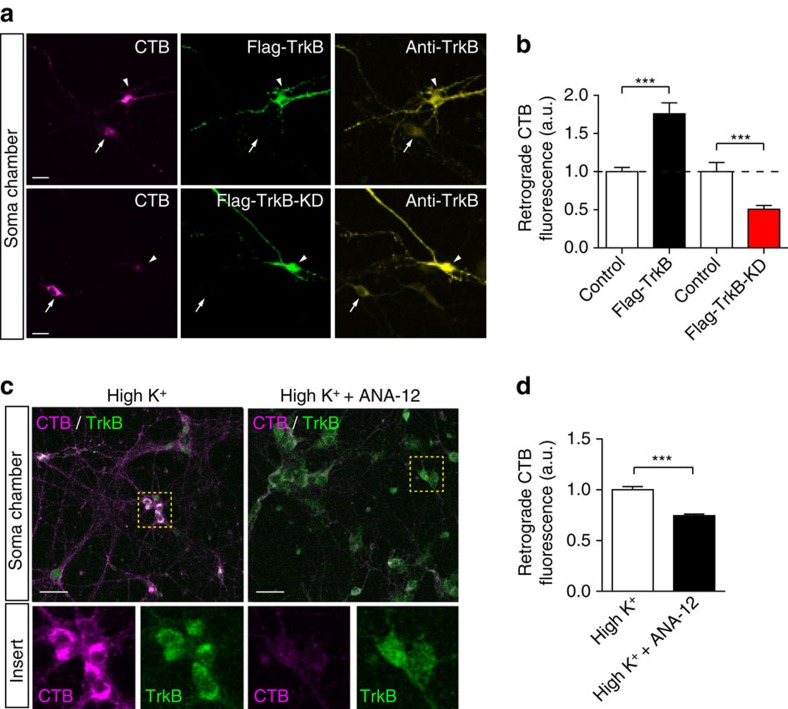
Genetic inhibition of TrkB activation prevents the activity-dependent retrograde transport of CTB. (**a**) DIV14–16 hippocampal neurons cultured in microfluidic chambers were transfected with Flag-TrkB-WT or Flag-TrkB-KD plasmids, and subsequently labelled with CTB under pulse-chased high K^+^. The fluorescence intensity of retrogradely transported CTB was observed with confocal microscopy in transfected neurons (arrow) and adjacent untransfected neurons (arrowheads) in the soma chambers only. Scale bar, 20 μm. (**b**) Quantification of **a** (mean±s.e.m., *n*=26, 46, 34 and 42 for the control, Flag-TrkB, control and Flag-TrkB-KD groups respectively, data from three independent cultures, ***P<0.001, Student’s *t*-test). (**c**) Neurons cultured in microfluidic chambers were labelled with CTB under high K^+^ pulse, then pretreated with 0.5 μM ANA-12 for 30 min. The fluorescence intensity of retrogradely transported CTB and endogenous TrkB were observed with confocal microscopy in the soma chambers. Scale bar, 50 μm. (**d**) Quantification of (**c**). (mean±s.e.m., *n*=15 and 17 for low K^+^ and high K^+^ groups, respectively, data from three independent cultures, ***P<0.001, Student’s *t*-test).

We next examined whether CTB retrograde trafficking was affected by overexpression of inactive, kinase-dead TrkB. Hippocampal neurons cultured in microfluidic chambers were transfected with a plasmid expressing wild-type TrkB (Flag-TrkB-WT) or kinase-dead TrkB (Flag-TrkB-KD), and labelled with CTB under high K^+^ stimulation for 5 min. We found that TrkB-WT transfection significantly increased the amount of retrogradely transported CTB in response to a 5 min pulse of stimulation (Fig. 8a,b). This demonstrates that TrkB-WT overexpression leads to an increase in the formation and retrograde transport of signalling endosomes. In sharp contrast, TrkB-KD expression significantly decreased the amount of CTB reaching the soma (Fig. 8a,b). A similar reduction in the overall retrograde transport of CTB was observed using ANA-12 (Fig. 8c,d). Taken together, these results suggest that TrkB activation is critically involved in controlling the flux of activity-induced signalling endosomes that undergo retrograde axonal transport and eventually reach the soma.

### Activity-dependent CTB carriers flux increase is BDNF independent

**Figure 9:**
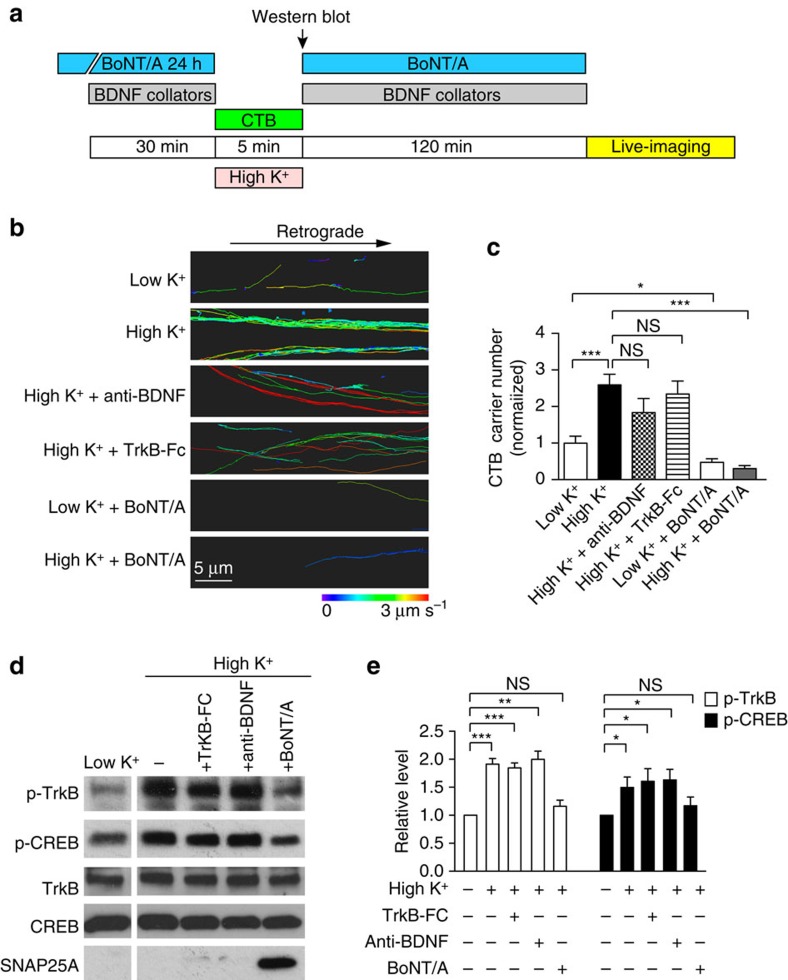
Activity-dependent CTB retrograde flux is dependent on synaptic activity but not endogenous BDNF secretion. (**a**) Hippocampal neurons cultured in microfluidic chambers were pretreated with 100 pM BoNT/A for 24 h or 20 μg ml^−1^ BDNF collators (BDNF blocking antibody; TrkB-Fc) for 30 min before 5 min CTB pulse in low or high K^+^ buffers as indicated. After wash-off, the chambers were chased in the original culture medium containing BoNT/A or BDNF collators for 2 h, then live-imaged with confocal microscopy. For western blotting, neurons were collected without the 2 h chase. (**b**) Imaris tracing of CTB tracks in representative live-imaging movies as treated in **a**, colour-coded average speed 0–3 μm s^−1^. Scale bar, 5 μm. (**c**) Number of retrograde CTB carriers with indicated treatments (mean±s.e.m., *n*=14, 31, 26, 13, 17 and 17 for low K^+^, high K^+^, high K^+^+anti-BDNF, high K^+^+TrkB-Fc, low K^+^+BoNT/A and high K^+^+BoNT/A, respectively, data are from three independent cultures, student’s *t-*test, *P<0.05; ***P<0.001; NS, not significant). (**d**) Western blot of treated hippocampal neurons showing that the high K^+^-induced increases in p-TrkB and p-CREB were not affected by BDNF collators but were abolished by BoNT/A treatment; total TrkB and CREB protein levels were used as controls. (**e**) Quantification of (**d**). (mean±s.e.m., *n*=3, data from three independent cultures, *P<0.05; **P<0.01; ***P<0.001; NS, not significant; Student’s *t*-test).

Finally, as synaptic activity also promotes BDNF secretion, which can subsequently act as a ligand to activate the TrkB pathway^34^,^35^, we investigated whether the coupling between synaptic activity and CTB retrograde flux could be mediated by BDNF-dependent TrkB activation. We used both a BDNF blocking antibody (anti-BDNF, 20 μg ml^−1^)^36^ and a chimeric BDNF scavenger (TrkB-Fc, 20 μg ml^−1^)^37^,^38^ to remove the endogenous BDNF secreted during stimulation, for 30 min before the pulse-chase (Fig. 9a). Although both BDNF collators effectively blocked the BDNF-dependent TrkB activation (Supplementary Fig. 3c), neither had any significant impact on the activity-dependent increase in CTB retrograde flux (Fig. 9b,c). Consistent with this, TrkB activation, as determined by high K^+^-induced TrkB phosphorylation, was also not affected by these BDNF collators (Fig. 9d,e; full scans in Supplementary Fig. 3d). This confirms our previous results, which demonstrated that the addition of BDNF had no effect on CTB retrograde flux (Supplementary Fig. 1d,e). Taken together, these results indicate that the activity-dependent increase in CTB retrograde flux is independent of BDNF.

We next determined whether CTB retrograde flux was affected on blockade of synaptic activity with botulinum neurotoxin type A (BoNT/A). BoNT/A acts by cleaving SNAP-25 and specifically prevents the fusion of synaptic vesicles with the presynaptic plasma membrane^39^,^40^. SNAP25 cleavage was confirmed using an antibody specifically designed to recognize BoNT/A-cleaved SNAP25 (SNAP25A)^41^,^42^ (Fig. 9d,e; Supplementary Figs 3d and 4). BoNT/A treatment prevented both the activity-dependent and basal level of CTB retrograde transport (Fig. 9b,c). BoNT/A also blocked the phosphorylation of TrkB and CREB elicited by depolarization (Fig. 9d,e). These results demonstrate that the induction of retrograde CTB flux is dependent on presynaptic activity.

## Discussion

In the present study, we demonstrate that the level of activity at the presynapse in hippocampal neurons, whether it be low or high activity, can induce changes in the frequency of CTB-positive retrograde carriers undergoing transport from the nerve terminal to the soma. We reveal that >50% of CTB carriers are TrkB-positive signalling endosomes, and that there is coupling between the level of presynaptic activity and the flux of signalling endosomes undergoing retrograde axonal transport. Given that the amount of activated TrkB per signalling endosome has been shown to be quantal in nature, it is likely that it is the number of signalling endosomes reaching the cell body that is responsible for monitoring the trophic response of each neuron. Importantly, we demonstrate that the transient TrkB activation that occurs when presynaptic activity is increased controls the activity-dependent increase in the flux of signalling endosomes in a BDNF-independent manner. This suggests that TrkB activation may play an active role in generating signalling endosomes with a retrograde fate independent of BDNF secretion. Finally, our SIM and morphometric analyses reveal a high number of sub-diffraction-limited CTB- and TrkB-positive carriers that may be involved in delivering the survival message.

CTB is a widely used neuroanatomical tracer with the ability to undergo retrograde transport in neurons^25^. We found that CTB enters nerve terminals in an activity-dependent manner, and following a 2–4 h chase, is found in retrograde axonal carriers that co-localize extensively with TrkB. Importantly, TrkB staining in resting conditions produced a low and surface-localized pattern, which is in sharp contrast with that observed in the CTB/TrkB vesicular carriers following stimulation. Considering that internalization of activated TrkB is elicited by neuronal activity in hippocampal neurons^10^, this suggests that the retrograde carriers containing both TrkB and CTB are signalling endosomes, destined to deliver a survival signal from the terminal to the soma. Our data are also consistent with the previous demonstration that CTB can be co-transported in the same retrograde carriers as the neurotrophin receptor p75^NTR^ and TrkB receptors^14^,^43^. The authors of these studies speculated that activated neurotrophin receptors internalized into terminals through different pathways may converge in the retrograde traffic and be packaged into ‘common’ compartments, presumably signalling endosomes^1^, which then undergo microtubule-dependent retrograde trafficking to induce signalling cascades and gene expression in the soma^2^,^4^,^9^,^44^.

The signalling endosome model has been proposed to describe the propagation of activated Trk signals from the axon terminal to the neuronal soma^1^,^2^. Although the presence of signalling endosomes is widely accepted, the precise nature(s) and regulation of these organelles remain unknown. In particular, it is not known how presynaptic activity translates into an increase in signalling endosome-encoded cell survival. Recent studies using super-resolution microscopy have shown that individual signalling endosomes appear to contain a limited number of signalling receptors^6^, suggesting that retrograde survival signalling could be quantal in nature. Consistent with this idea, recent analysis has demonstrated that signalling endosomes have a fixed number of phosphorylated tyrosine receptor kinases^13^. Our findings suggest that it is the flux of signalling endosomes reaching the cell body that encodes the level of presynaptic activity, and translates this into a survival signal. Our data reveal a clear increase in the frequency of retrograde carriers delivering both CTB and TrkB from the nerve terminal to the cell soma following a short, transient burst of activity, whereas BoNT/A pretreatment not only completely blocked this increased flux despite equivalent stimulation but also lowered the basal level of retrograde CTB flux in resting conditions. These results strongly support the hypothesis that the soma of a neuron with lower synaptic activity receives fewer signalling endosomes than that of a cell with high synaptic activity, with ramifications for survival^45^,^46^,^47^,^48^,^49^,^50^.

Previous studies have clearly established that there is constitutive delivery of retrogradely transported neurotrophins and their receptors in different types of primary neurons^1^,^4^,^6^,^43^,^51^. To the best of our knowledge, our study is the first to reveal a coupling between synaptic activity and the number of retrograde signalling endosomes in both hippocampal neurons and motor neurons from *D. melanogaster* larvae, suggesting that this coupling represents a conserved regulatory mechanism.

Genetic manipulations in *Drosophila* allowed us to chronically increase motor neuron activity, thereby demonstrating a correlation with increased retrograde signalling endosome transport. Retrograde axonal transport has previously been reported in *Drosophila* larval motor neurons^52^,^53^. However, this process is not regulated by the activation of TrkB receptors in flies, but by neurotrophin-type factors, such as BMP^54^ P150 (ref. 55) and Toll^56^. Investigating the respective contributions of each of these mechanisms will be required to unravel the mechanism(s) controlling the coupling between synaptic activity and the flux of retrogradely transported signalling endosomes.

Clathrin-mediated endocytosis and bulk endocytosis are the two main modes for synaptic vesicle retrieval^21^,^57^. On the basis of our data, internalized CTB is sorted into both synaptic vesicles and larger endosomes following high K^+^-induced synaptic activation. These are therefore likely to be the source of CTB-positive signalling endosomes. Indeed, we noted that, following a 4 h chase, the number of CTB-labelled endosomes present in nerve terminals was clearly reduced, suggesting that they could contribute to the generation of retrograde carriers leaving the terminals. More work is needed to pinpoint the actual contribution of the different presynaptic endocytic pathways in the process of generating signalling endosomes and recycling synaptic vesicles. Using electron microscopy and super-resolution SIM on the unidirectional axon bundles, we found a high proportion of CTB-positive retrograde carriers with a diameter <150 nm, indicating that these may constitute a significant source of the signalling endosomes that reach the soma. Interestingly, we also found that a number of these small CTB retrograde endosomes closely aligned with each other to form consecutive structures. These were reminiscent of single-molecule quantum dot BDNF-labelled compartments, previously described as elongated multiple vesicular bodies^58^. Several different types of organelles contain endocytosed neurotrophin receptors, including electron-lucent endosomes ranging from 50 nm to >200 nm in diameter^6^,^51^,^59^ and multivesicular bodies^60^,^61^. Our results further confirm this morphological heterogeneity of signalling endosomes. Interestingly, they also show that the sub-diffraction-limited pool of retrograde carriers is increased by a pulse of stimulation. A future challenge will be to fully define these compartments, including their cargo, their regulation and their potential role in neuronal survival.

The activity-dependent increase in the flux of CTB retrograde carriers is similar to that observed with autophagosomes undergoing axonal retrograde transport^16^. Genetic and pharmacological inhibition of TrkB prevented the activity-dependent increase in signalling endosomes but did not affect the number of autophagosomes undergoing retrograde transport. This result suggests a limited cross-link between these two pathways, and indicates the existence of a TrkB-dependent sorting mechanism that is selective for the generation of retrograde signalling endosomes and is upregulated by synaptic activity.

Although TrkB activation was required to couple synaptic activity with the flux of retrograde CTB carriers, we found that BDNF collators (anti-BDNF blocking antibody and TrkB-Fc), as well as exogenously applied BDNF, failed to affect this coupling. These results suggest that BDNF may not be required to promote this pathway. First, we could speculate that increased neuronal activity alone promotes the secretion of alternative TrkB activators, such as NT4. In addition, considering that two different BDNF receptors, p75^NTR^ and TrkB, have been reported to have opposing actions in neurons^62^, our observation that BDNF collators have no effect on retrograde transport or the phosphorylation of TrkB could be explained by the fact that these collators alleviate an inhibitory effect of p75^NTR^ signalling on TrkB activation. Third, it is possible that TrkB receptors could be indirectly transactivated by adenosine^63^,^64^ or by the MAPK pathway, similar to other tyrosine kinase receptors^63^. However, further experiments are required to pinpoint the precise cascade of molecular events leading to the encoding of the level of synaptic activity by the flux of retrograde signalling endosomes in a BDNF-independent and TrkB activation-dependent pathway.

Our data show that internalized CTB is sorted into both synaptic vesicles and larger endosomal structures reminiscent of bulk endosomes as described previously^18^. Bulk endosomes are therefore likely to contribute to the sorting events that lead to the generation of CTB-positive signalling endosomes. Indeed, our data demonstrate that, in response to stimulation, the number of nerve terminals containing bulk endosomes more than doubles, as does the number of endosomes per terminal. More strikingly, after a chase of 4 h, the number of bulk endosomes returns to control levels, suggesting that sorting has taken place and/or that these large endosomes have undergone retrograde trafficking. In support of this view we observed larger CTB-positive structures inside the axon by electron microscopy. Bulk endocytosis is therefore likely to be a key element of this pathway, not only by budding small synaptic-like vesicles in nerve terminals^65^, but also by generating large endosomes that undergo retrograde trafficking. More work is needed to pinpoint the actual contribution of bulk endocytosis in the process of generating signalling endosomes and recycling synaptic vesicles. The fact that synaptic activity increases the flux of CTB retrograde carriers advocates for the presence of a number of regulatory mechanisms that effectively couple these two pathways. Whether both recycling synaptic vesicles and signalling endosomes bud from the same bulk endosomes will require further investigation.

In summary, we have uncovered a coupling between synaptic activity, trophic signalling and the generation of axonal retrograde signalling endosomes, which contain TrkB receptors and nerve terminal-derived CTB as cargo, and have characterized their kinetic and morphological nature. Our data reveal a novel role of presynaptic activity in controlling the flux of retrograde signalling endosomes that will eventually reach the neuronal cell body and deliver cell survival signals. These results suggest that the flux of signalling endosomes undergoing axonal transport constitutes a digital output of the level of synaptic activity experienced by nerve terminals. How the flux of signalling endosomes reaching the cell body is decoded to pursue or halt a survival signal warrants further investigation.

## Methods

### Antibodies fluorescent labels and reagents

Cholera toxin subunit-B (recombinant) labelled with Alexa Fluor 488 or 555 was obtained from ThermoFisher Scientific (#c-34775, #c-34776). Mouse anti-synaptobrevin-2 (VAMP2) antibody was obtained from Synaptic Systems (#104 211, 1:2,000 dilution) and rabbit anti-TrkB polyclonal antibody from Biosensis (#R-121-100, 1:2,000 dilution). Rabbit monoclonal antibody against phospho-TrkB (Tyr707/706) came from Cell signalling Technology (#4621, 1:500 dilution), anti-CREB 1 rabbit polyclonal antibody from Santa Cruz Biotechnology (#sc-186, 1:500 dilution), mouse monoclonal antibody against phosphor-CREB (Ser133) from Cell signalling Technology (#9196, 1:500 dilution), and mouse anti-β-tubulin III from Covance (catalog #MMS-435P, 1:2,000 dilution), BoNT/A-truncated SNAP25 antibody (1:500 dilution) was a kind gift from D. Sesardic, Division of Bacteriology, National Institute for Biological Standards and Control, Hertfordshire, UK. Recombinant human BDNF (#b3795) was from Sigma-Aldrich. BDNF blocking antibody was from DSHB (BDNF-#9 bioreactor supernatant, 20 μg ml^−1^). Recombinant Human TrkB Fc Chimera Protein was from R&D Systems (#688-TK, 20 μg ml^−1^). Alexa Fluor secondary antibodies and maleimide-reactive fluorophores were purchased from Life Technologies, and used at 1:1,000 dilutions. The remaining reagents were obtained from Electron Microscopy Sciences or Sigma Aldrich unless otherwise specified.

### Neuronal cultures

Hippocampal neurons were cultured from embryonic age 18 (E18) Sprague Dawley rats or C57BL/6 mice. The Animal Ethics Committee at the University of Queensland approved all the experimental procedures (permit number is QBI/313/13/NHMRC). For non-microfluidic experiments, mouse hippocampal neurons were plated on coverslips for confocal microscopy or plastic dishes for electron microscopy, with the astroglia seeded on the surrounding plastic as previously described^66^. For experiments performed with microfluidic chambers, rat hippocampal neurons were prepared as described previously^16^ and seeded in microfluidic chambers (Xona, #RD450) following the manufacturer’s protocol^19^. For the SIM super-resolution experiments, cultures in microfluidic devices were prepared from E18 rat brain and digested with 0.25% trypsin-EDTA for 20 min at 37 °C followed by trituration with pipettes in the plating medium (DMEM with 10% fetal bovine serum and 10% F12), and then plated onto coverslips coated with poly-D-lysine. After culturing for 24 h, the medium was changed to neuronal culture medium (Neurobasal medium containing 1% glutamate and 2% B27).

### Live imaging of CTB retrograde carriers

Stimulation and labelling were carried out on rat hippocampal neurons cultured in microfluidic chambers between days *in vitro 14* (DIV14) and DIV17. As illustrated in Fig. 2a, culture medium was removed from all chambers and neurons were incubated for 5 min at 37 °C in low K^+^ buffer (15 mM HEPES, 145 mM NaCl, 5.6 mM KCl, 2.2 mM CaCl_2_, 0.5 mM MgCl_2_, 5.6 mM D-glucose, 0.5 mM ascorbic acid, 0.1% bovine serum albumin (BSA), pH 7.4) or high K^+^ buffer (the same as the low K^+^ buffer except that it contained 95 mM NaCl and 56 mM KCl), with 50 ng ml^−1^ CTB-Af488 or CTB-Af555 added to the nerve terminal chambers only. After washing with low K^+^ buffer, neurons were returned to the original conditioned growth medium for 2 h before imaging. Axon channels were visualized in live-imaging buffer (25 mM HEPES, 145 mM NaCl, 5.6 mM KCl, 0.5 mM MgCl_2_, 2.2 mM CaCl_2_, 5.6 mM D-glucose, pH 7.4). For instant speed analysis, time-lapse images of CTB were acquired with a Zeiss LSM710 inverted microscope maintained at 37 °C and 5% CO_2_ at 0.1–0.3 s intervals for 95 s. CTB carriers were tracked automatically with the ‘spot’ function of Imaris software (Imaris7.7, Bitplane), with the same parameter settings being used for all groups. For frequency and average speed analysis, time-lapse images of CTB carriers were captured with a Zeiss LSM510 inverted microscope at 0.4 s intervals for 400 s and tracked with Imaris, after which the frequency, average speed and dwelling ratio of CTB tracks with duration time ≥20 s were compared between groups. Kymographs were generated using ImageJ software (NIH) using the plugin Multi-Kymograph for ImageJ.

### Confocal microscopy

For immunofluorescence microscopy of fixed cells, microfluidic devices were removed and neurons fixed for 2–4 h with PBS containing 4% paraformaldehyde and 4% sucrose, then processed for immunocytochemistry as previously described^67^. Permeabilization was performed using 0.1% saponin, 0.2% gelatin and 1% BSA in PBS. Imaging was carried out on a Zeiss LSM 710 confocal microscope and analysed with Zen (Zeiss) and ImageJ software. Mouse hippocampal neurons cultured on glass coverslips were treated in the same manner, except that 50 ng ml^−1^ of CTB-Af555 was added to the buffer for 5 min before fixation with 4% paraformaldehyde and processing for immunocytochemistry as previously described^66^. Imaging was carried out on a Zeiss LSM 510 inverted confocal microscope and analysed with Zen (Zeiss) and ImageJ software. All images were compiled using Illustrator CS 5.1 (Adobe). For analysis of immunofluorescence images from confocal microscopy, 3–5 different neuronal soma were selected from different positions in each field (394 μm × 394 μm), and 8–9 fields from three independent cultures were used per analysis. The morphology of the soma was derived from masks using the β-Tub III channel, and the fluorescence intensity of the masked regions was measured using Image J plugins.

### Structured illumination microscopy

DIV14 neurons cultured in microfluidic chambers were fixed and immunostained with TrkB and β-tubulin III antibodies before being imaged with structured illumination microscopy (SIM; ELYRA PS1, Zeiss) equipped with a × 63 objective (α Plan-Apochromat × 63/1.46 oil-immersion) and a PCO scientific CMOS camera. Images were obtained with a SIM grating size of 34–51 μm at different wavelengths, and using five rotations. Structured illumination images were then aligned and processed using Zen software. The estimated lateral resolution limit was 82 nm. Image J was used to analyse the co-localization rate of SIM images; 5–6 none-overlapping regions of interest (13 μm × 10 μm) were selected along each axon channel field (length≈80 μm; width ≈10 μm ), and 8–9 fields from three different neuronal cultures were used. The co-localization rates between different channels were calculated as the Manders coefficient using the JACoP plugin^68^ of ImageJ software (NIH). CTB particles with a diameter <150 nm were quantified with Imaris (Bitplane) software.

### Electron microscopy

Mouse hippocampal neurons (DIV 14–16) in 3 cm plastic dishes were incubated with 10 μg ml^−1^ CTB-HRP in either high K^+^ or low K^+^ buffer for 5 min. Cells were subsequently either washed and incubated in culture medium for a further 4 h before fixation, or washed in PBS and directly fixed. Rat hippocampal neurons cultured in microfluidic devices (DIV 14–17) were treated as described for confocal microscopy, except that 10 μg ml^−1^ CTB-HRP was added to the nerve terminal chambers for the period of stimulation. Cells were returned to growth medium for 4 h before fixation. All cells were fixed in 2.5% glutaraldehyde for 24 h. Following fixation, cells were processed for 3,39-diaminobenzidine (DAB) cytochemistry using standard protocols. Fixed cells were contrasted with 1% osmium tetroxide and 4% uranyl acetate before dehydration and embedding in LX-112 resin^66^. Sections (∼50 nm) were cut using an ultramicrotome (UC64; Leica). To quantify CTB-HRP endocytosis, presynaptic regions were visualized at × 60,000 using a transmission electron microscope (model 1011; JEOL) equipped with a Morada cooled CCD camera and the iTEM AnalySIS software. Membrane-bound compartments within the presynaptic region (co-cultured cells) or within the cell soma proximal region of the microfluidic channel were counted and scored for the presence or absence of the DAB reaction product, and the maximum diameter was measured using ImageJ software.

### *D. melanogaster* larvae experiments

*D. melanogaster* stocks were maintained at 22 °C on standard yeast-based *Drosophila* medium, on a 12 h light–12 h dark cycle To chronically increase neuronal activity levels in larval motor neurons, the *C380-Gal4* driver was used to drive a UAS line carrying transgenes encoding dominant-negative forms of the K^+^ channels, ether-à-go-go (Eag) and Shaker (Sh) (2nd chromosome). The UAS line was generated by recombining the *UAS-eag*^*Δ932*^ (ref. 69), and *UAS-SDN*^70^ lines (Gift from Dr Subhabrata Sanya, Emory University, USA). Transgenic larval offspring (designated *eag, Sh*) were generated by crossing male Gal4 flies with virgin UAS females. For control larvae, C380-Gal4 male flies were crossed with w^118^ virgin females (the background strain for C380-Gal4 flies). As previously described, wandering third instar larval offspring were dissected and pinned out on a Sylgard-lined dish containing Schneider's insect medium^24^. In the dissected offspring, the ventral nerve cord was left intact. For retrograde transport experiments, CTB-Af488 was added to the preparation to a final concentration of 2 μg ml^−1^ and incubated at room temperature for 2 h. Time-lapse imaging of CTB retrograde transport in motor neuronal nerve segments innervating larval wall muscles 5 and 8 of abdominal segments 2 and 3 was performed on a Zeiss LSM 510meta upright confocal microscope equipped with a × 63 water-dipping objective (numerical aperture 1.4). For central nervous system accumulation experiments, larval preparations were incubated with 2 μg ml^−1^ CTB-Af555 for 4 h before imaging. Confocal Z-stack images of CTB puncta accumulated within the ventral nerve cord were acquired. Images and time-lapse sequences were processed and analysed using ImageJ software (NIH).

### Data availability

The data that support the findings of this study are available on request from the corresponding author F.A.M.

## Additional information

**How to cite this article:** Wang, T. *et al*. Flux of signalling endosomes undergoing axonal retrograde transport is encoded by presynaptic activity and TrkB. *Nat. Commun.* **7,** 12976 doi: 10.1038/ncomms12976 (2016).

## Supplementary Material

Supplementary Information Supplementary Figures 1-4

Supplementary Movie 1 Retrograde flux of CTB carriers in resting neurons

Supplementary Movie 2 Retrograde flux of CTB carriers in stimulated neurons

Supplementary Movie 3 Retrograde flux of CTB carriers in stimulated neurons pretreated with ANA-12

Supplementary Movie 4 Retrograde flux of CTB carriers in stimulated neurons pretreated with K252a
