# Corrigendum: Flux of signalling endosomes undergoing axonal retrograde transport is encoded by presynaptic activity and TrkB

**DOI:** 10.1038/ncomms13768

**Published:** 2016-11-23

**Authors:** Tong Wang, Sally Martin, Tam H. Nguyen, Callista B. Harper, Rachel S. Gormal, Ramon Martínez-Mármol, Shanker Karunanithi, Elizabeth J. Coulson, Nick R. Glass, Justin J. Cooper-White, Bruno van Swinderen, Frédéric A. Meunier

Nature Communications 7: Article number: 12976 doi: ; DOI: 10.1038/ncomms12976 (2016); Published: 11 23 2016; Updated: 11 23 2016

In Fig. 3 of this Article, an image attribution was inadvertently omitted. The drawing of a *Drosophila* larva in Fig. 3a is reproduced from the website http://www.prokop.co.uk/Research/Drosi-Info/nerve-cords.html with the kind permission of Dr Andreas Prokop.

