# Supplementary material for: Flux of signalling endosomes undergoing axonal retrograde transport is encoded by presynaptic activity and TrkB
Source: Nat Commun. 2016 Sep 30;7:12976. doi: 10.1038/ncomms12976 (PMC5427517; doi:10.1038/ncomms12976)
Supplement: Supplementary Information — Supplementary Figures 1-4 [file ncomms12976-s1.pdf]

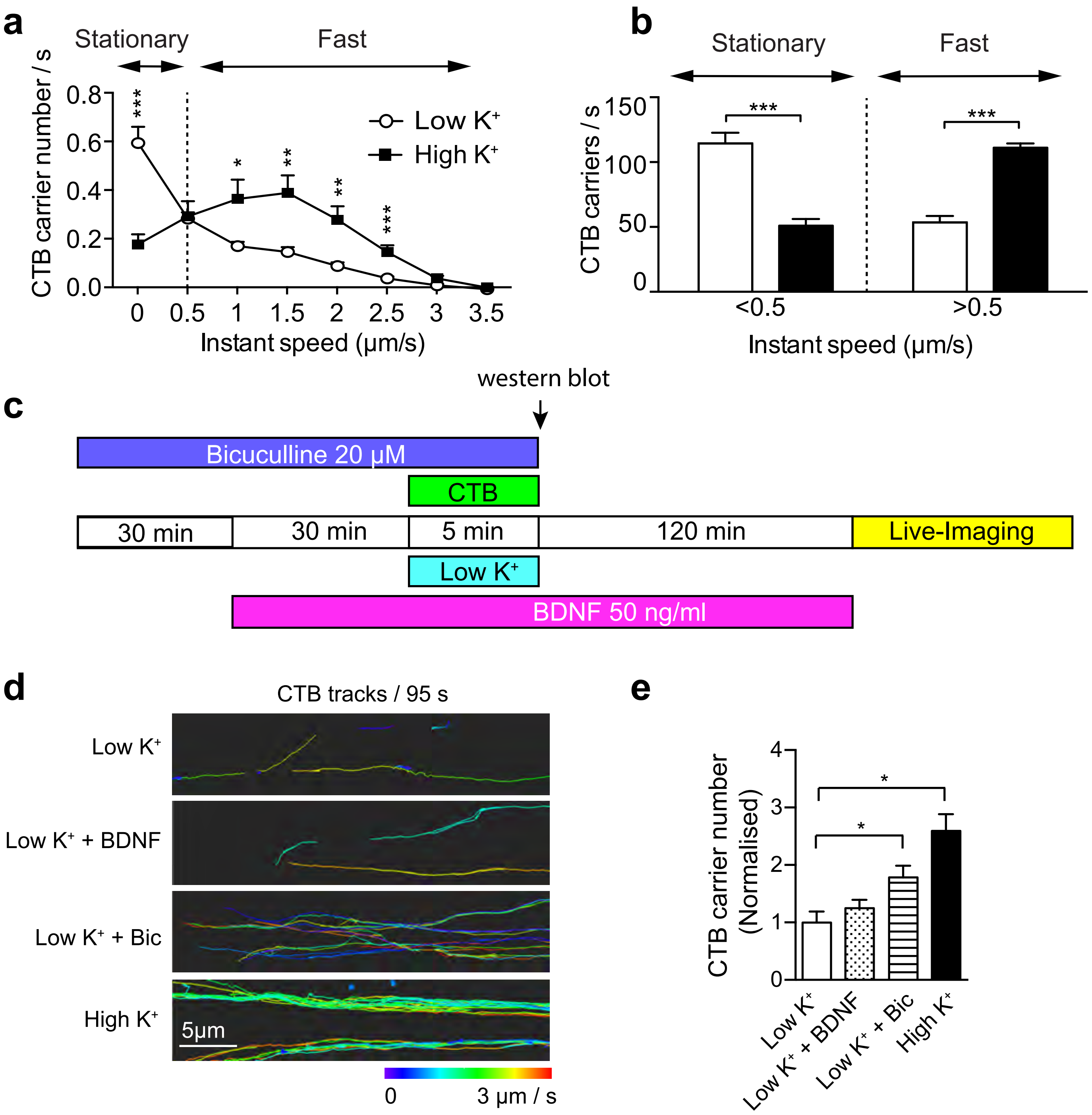

Supplementary Fig. 1 CTB retrograde flux is up-regulated by synaptic activity but not BDNF.

(a) Frequency distribution of the instant speed of axonal retrograde CTB carriers in low K^+- and high K^+-treated neurons, showing a significant decrease in the frequency of stationary carriers and a significant increase in the frequency of fast axonal carriers. **(b)** Grouped analysis of instant speeds of stationary or fast moving CTB carriers, showing a significant difference between low K^+- and high K^+- treated neurons (mean±s.e.m, n=24 and 30 for low K^+ and high K^+ respectively, data are from 4 independent preparations, Student's *t*-test, n.s., not significant, *$p<0.05$, **$p<0.01$, ***$p<0.001$). **(c)** Hippocampal neurons cultured in microfluidic chambers were pretreated with 50 ng/ml BDNF or 20 µg/ml bicuculline for the indicated time before being pulsed at 37°C for 5 min with CTB in low- or high-K^+ buffers. After wash-off, the chambers were chased in the original culture medium for 2h, then live-imaged by confocal microscopy. **(d)** Imaris tracing of CTB tracks in representative movies (average speed of 0-3 µm/s was color-coded). Bar=5 µm. **(e)** Number of retrograde CTB carriers after treatment with BDNF (50 ng/ml), bicuculline (20 µg/ml), low K^+ and high K^+. (mean±s.e.m, n=14, 16, 29 and 31 for low K^+, low K^++BDNF, low K^++bicuculline and high K^+ respectively, data from 3 independent cultures, Student's *t*-test, *$p<0.05$).

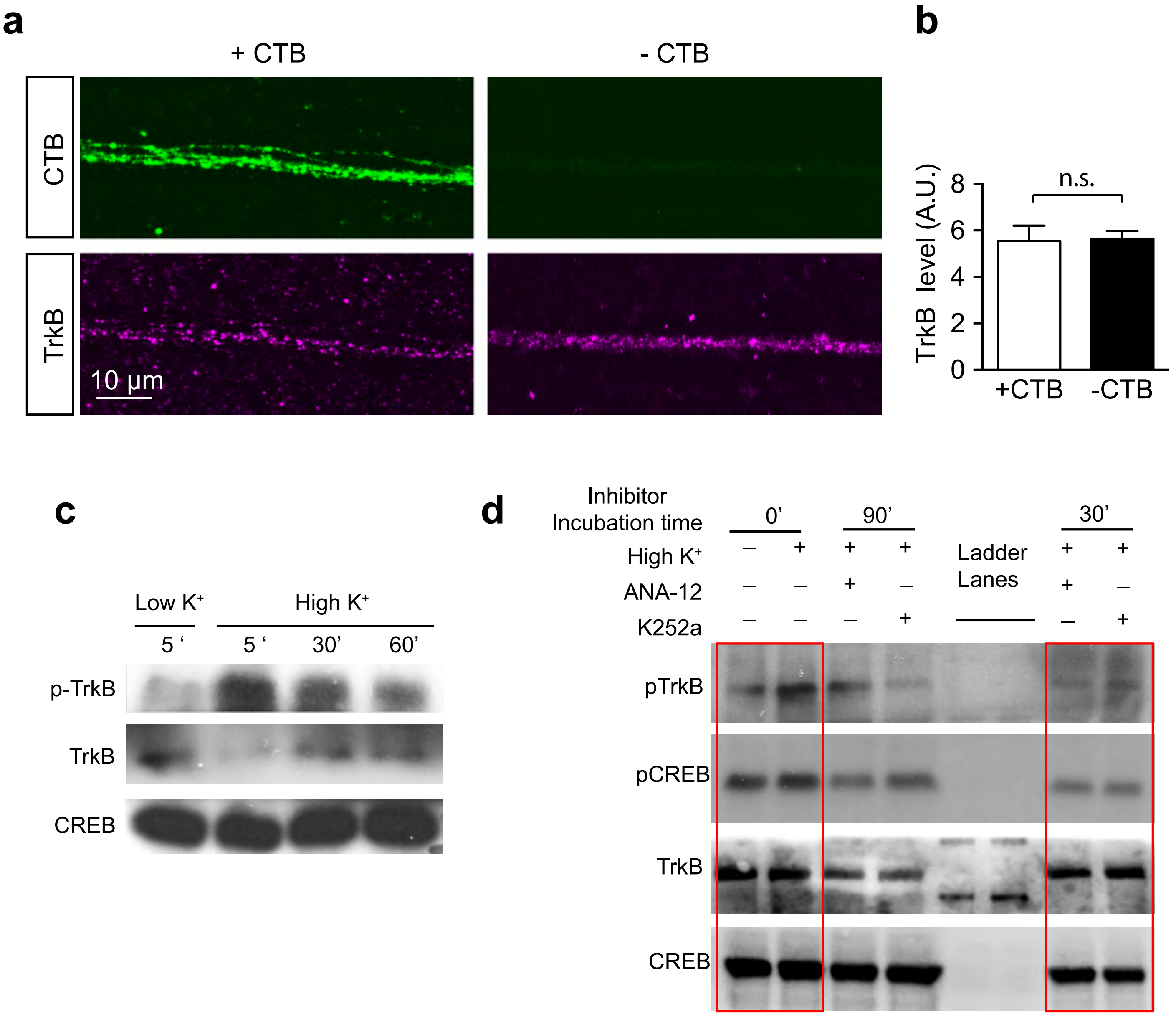

Supplementary Fig. 2 Kinetic analysis of CTB carriers after TrkB inhibitor pretreatment. (a) Representative images of the endogenous TrkB level along axons in microfluidic chambers labeled with or without CTB under 5 min high K^+ pulse. TrkB antibody was used to label endogenous TrkB receptors. Bar=10 µm. **(b)** The endogenous TrkB level was not affected by CTB labeling. (mean ± s.e.m, n=18 (-CTB) and 23 (+CTB) channels, data from 3 independent neuron cultures, n.s. no significant difference; Student's *t*-test). **(c)** Western blot showing that the p-TrkB level peaked 5 min after the high K^+ pulse and then gradually decreased. **(d)** Full lane scans of Fig. 5f .

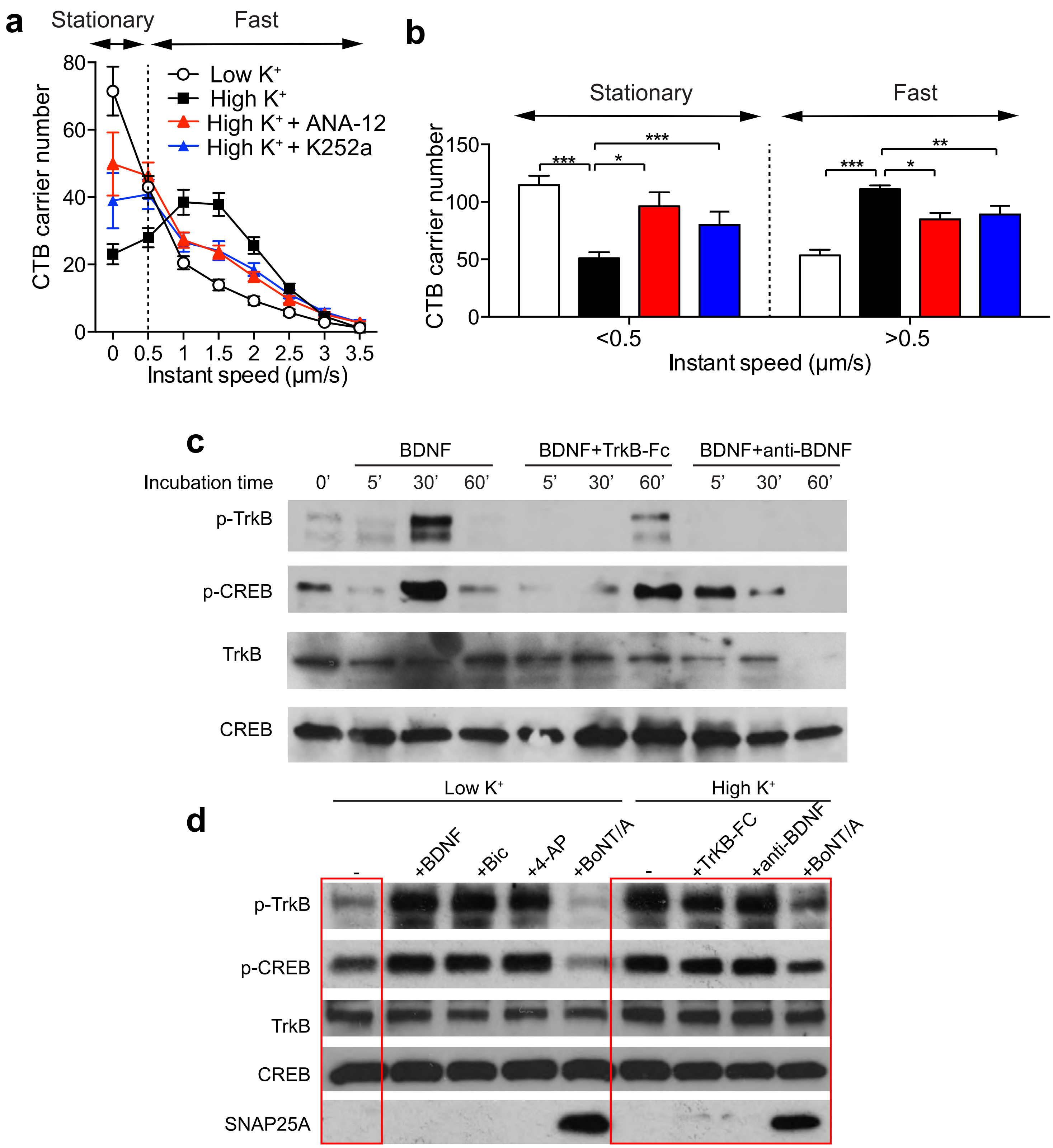

Supplementary Fig. 3 Synaptic activity induced TrkB activation is blocked by BoNT/A but not BDNF collators. (a) The reduced peak of CTB carriers with high instant speeds, and the increased peak of those with low instant speeds are shown. The increased number of stationary carriers (speed < 0.5 μm/s) and the decreased number of fast carriers (speed > 0.5 μm/s) are shown in **(b)**. (mean ± s.e.m, n= 78 (low K^+), 81 (high K^+), 83 (high K^++ANA-12) and 45 (high K^++K252a) tracks, data from 3 independent neuron preparations, *p<0.05, **p<0.01,***p<0.001, Student's t-test)**. (c)** The effectiveness of BDNF collators was examined by blocking the p-TrkB increase induced by BDNF. After 2 h of serum starvation, 20 μg/ml TrkB-Fc or 20 μg/ml BDNF blocking antibody was added to the culture medium together with 50 ng/ml BDNF, and the cultures were incubated for the indicated time periods before collection and western blot. Control is from cultures with serum starvation only. **(d)** Full lane scans of Fig. 9d.

sFigure-4 Meunier

Fig. 5f and sFig. 2d

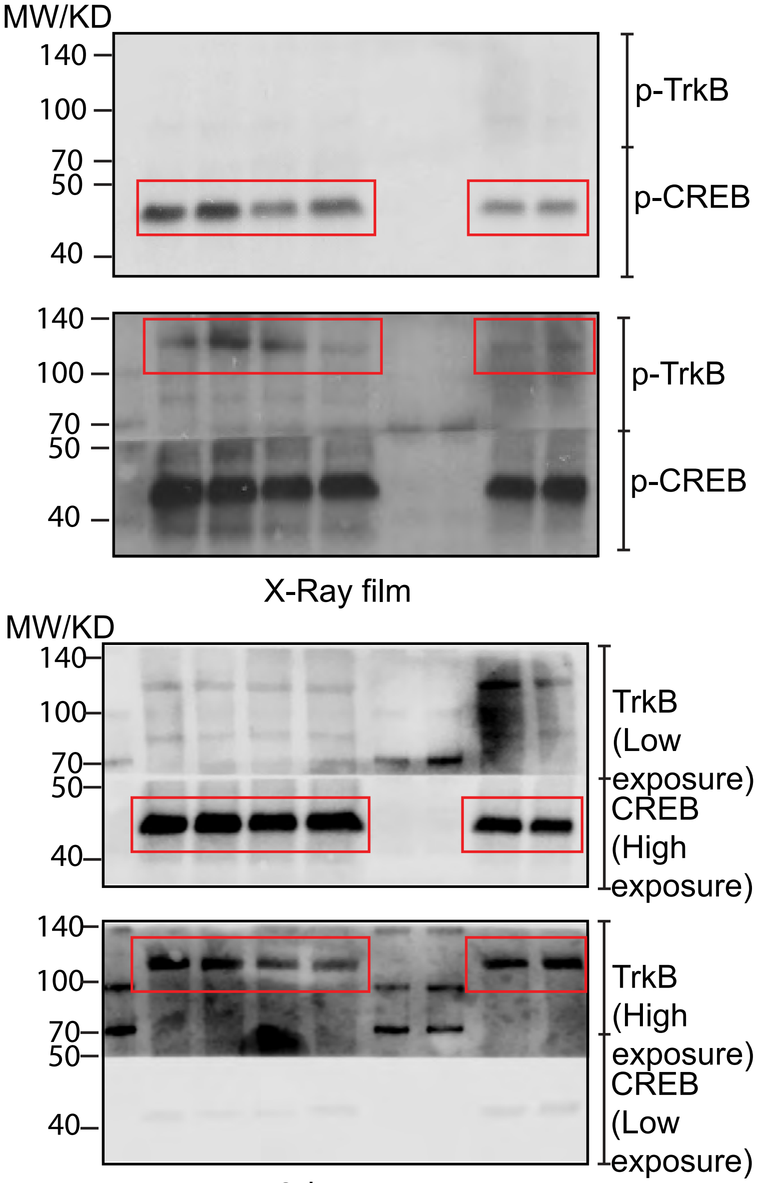

Odyssey Imager

sFig. 2c

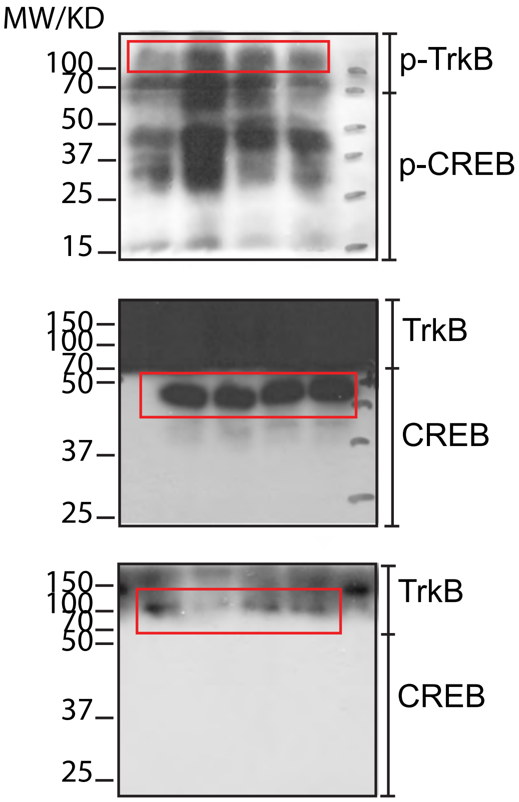

X-Ray film

Fig. 9d and sFig. 3d

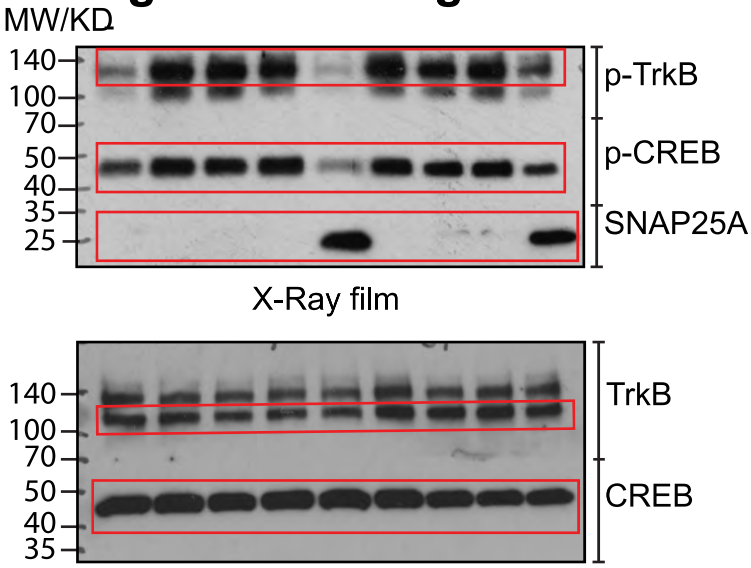

X-Ray film

sFig. 3c

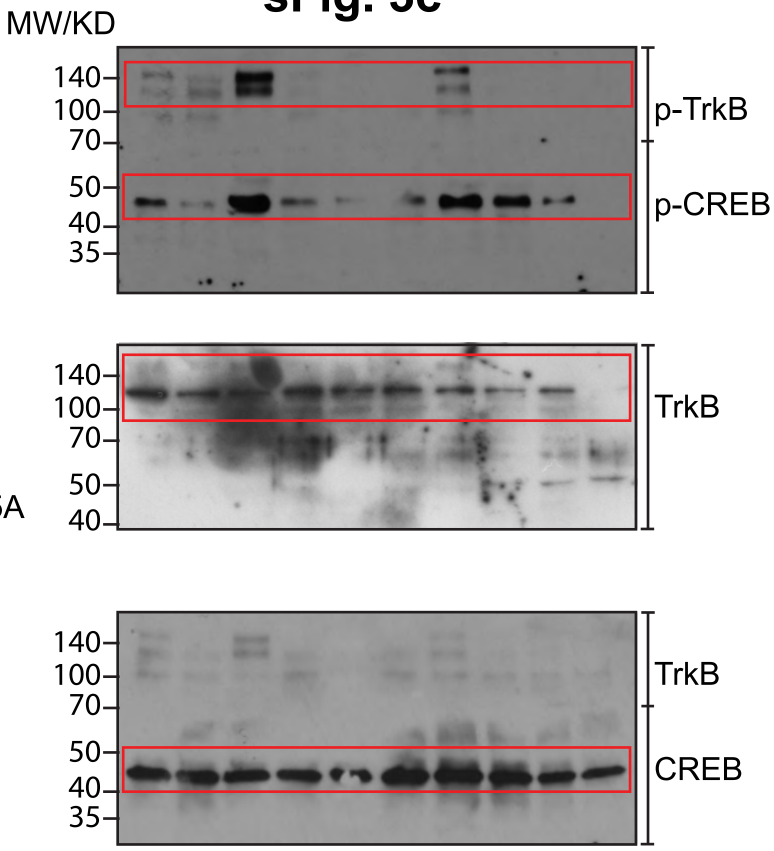

X-Ray film

Supplementary Fig. 4: Full scans data of western blots.
